# Trends in the annual incidence of carbapenem-resistant *Klebsiella pneumoniae* bloodstream infections: a 8-year retrospective study in a large teaching hospital in northern Italy

**DOI:** 10.1186/s12879-015-1152-0

**Published:** 2015-10-13

**Authors:** Cristiano Alicino, Daniele Roberto Giacobbe, Andrea Orsi, Federico Tassinari, Cecilia Trucchi, Giovanni Sarteschi, Francesco Copello, Valerio Del Bono, Claudio Viscoli, Giancarlo Icardi

**Affiliations:** Department of Health Sciences, University of Genoa and Hygiene and Infection Control Unit, IRCCS AOU San Martino-IST teaching Hospital, Genoa, Italy; Department of Health Sciences, University of Genoa and Infectious Diseases Unit, IRCCS AOU San Martino-IST teaching Hospital, L.go R. Benzi, 10-16132 Genoa, Italy; Occupational Medicine Unit, IRCCS AOU San Martino-IST teaching Hospital, Genoa, Italy

**Keywords:** Incidence, Bloodstream infection, Carbapenem-resistant *Klebsiella pneumoniae*

## Abstract

**Background:**

Bloodstream infections (BSI) due to carbapenem-resistant (C-R) *Klebsiella pneumoniae* (Kp) are of global concern from both clinical and public health standpoints. This retrospective study aimed to describe C-R Kp BSI epidemiology in a large teaching hospital in northern Italy.

**Methods:**

Between 1 January 2007 and 31 December 2014, annual incidences both of C-R Kp BSI and of carbapenem-susceptible (C-S) Kp BSI were calculated as the number of events per 10,000 patient-days. A Chi square test for linear trend was used to assess the change in the incidence of C-R Kp BSI and C-S Kp BSI over the study period. Crude 30-day mortality rates were provided both for C-R Kp BSI and for C-S Kp BSI.

**Results:**

From 2007 to 2014, we observed 511 episodes of Kp BSI, 349 of which were caused by C-R Kp (68.3 %). The incidence of C-R Kp BSI considerably increased from 0.04/10,000 patient-days in 2007 to 1.77/10,000 patient-days in 2014 (Chi square for trend *p* < 0.001). The highest incidence of C-R Kp BSI was observed in intensive care units (ICUs), with a peak of 22.01 C-R Kp BSI/10,000 patient-days in 2012. A less marked but significant increase of C-S Kp BSI was also observed (Chi square for trend *p* = 0.004). Crude 30-day mortality was 36.1 % in patients with C-R Kp BSI and 23.5 % in those with C-S Kp BSI.

**Conclusions:**

During the study period, we observed a dramatic increase in the incidence of C-R Kp BSI in our hospital. More concerted infection-control efforts are needed to contain this alarming C-R Kp diffusion.

## Background

Bloodstream infections (BSI) due to carbapenem-resistant (C-R) *Klebsiella pneumoniae* (Kp) are of global concern from both clinical and public health standpoints. Indeed, these severe infections are caused by rapidly spreading clones that are frequently resistant to almost all the antibiotics used in the everyday clinical practice [1–6].

Occurring especially across Southern Europe, the rapid diffusion of C-R Kp has worryingly impressed the medical community, and several guidelines have been recently developed to reduce the spread of these difficult-to-treat gram-negative rods [7–9]. In Italy, carbapenem resistance among blood Kp isolates has dramatically increased from 1.3 % in 2009 to 29.1 % in 2012, and even higher rates of 34.3 % have been reported in 2013 [10, 11]. With the aim of more clearly investigate this alarming C-R Kp diffusion, we retrospectively assessed the epidemiology of C-R Kp BSI in a large teaching hospital in northern Italy over a 8-year period.

## Methods

### Data collection and definitions

We performed a retrospective study in IRCCS AOU San Martino – IST, a 1,300-beds tertiary adult acute-care teaching hospital in Genoa, Italy. Between 1 January 2007 and 31 December 2014, numbers of hospital patient-days were obtained from the digital archives of patients’ clinical charts. Then, numbers of both carbapenem-susceptible (C-S) BSI and C-R Kp BSI were identified through the computerized microbiology laboratory database. The completeness and quality of laboratory data was successfully assessed by matching the hospital admission code with data obtained by clinical charts.

Only health-care associated C-R Kp BSI were considered for the analysis. According to the European Center for Disease Control and prevention (ECDC) definitions, a health-care associated C-R Kp BSI was defined as a positive blood culture collected at least 48 h after hospital admission, or within 48 h from hospital admission in those patients who had been discharged in the preceding two days [12]. For patients with multiple episodes of C-R Kp BSI, a novel event was considered as independent if occurring at least 30 days after the last positive blood culture [12].

This epidemiological analysis was performed within the Institutional surveillance of C-R Kp BSI that is periodically reported to the Regional Health Authority as a component of the Regional Plan for the Healthcare-associated infections Prevention and Control, approved by regional and national laws [13, 14]. The study involved the analysis of existing anonymized clinical and laboratory data. An informed consent for the use of anonymized data for scientific purposes is signed by all patients admitted to IRCCS AOU San Martino – IST and included in surveillance databases. The study has been approved by the Regional Ethics Committee of Liguria Region.

### Microbiology

The Vitek 2 system (bioMérieux, Marcy l’Etoile, France) was used for Kp identification and antimicrobial susceptibility testing. The interpretative breakpoints were based on the European Committee on Antimicrobial Susceptibility Testing (EUCAST) criteria (EUCAST breakpoint tables for interpretation of MICs and zone diameters, version 4.0, 2014; http://www.eucast.org). For the analysis of data, Kp isolates which were resistant to one or more carbapenems tested in our institution (i.e., ertapenem, imipenem, or meropenem) were considered to be C-R, while isolates showing full or intermediate susceptibility to all tested carbapenems were classified as C-S.

### Statistical analysis

The primary study analysis aimed to establish the annual incidences of C-R Kp BSI during the study period. Annual incidences of C-R Kp BSI with their 95 % confidence intervals (CI) were calculated as the number of events per 10,000 patient-days. In addition, annual incidences of C-R Kp BSI were also stratified by subgroups according to the ward where the diagnosis of C-R Kp BSI was made (i.e., intensive care units [ICUs], medical wards, surgical wards, or rehabilitation wards). A Chi square test for linear trend was used to assess the change in the incidence of C-R Kp BSI in our hospital over the study period.

An additional aim was to detail the overall trends in the incidence of Kp BSI. Therefore, annual incidences of health-care associated C-S Kp BSI were also calculated, by means of the methods described above. Finally, crude 30-day mortality rates were assessed both for C-R Kp BSI and for C-S Kp BSI.

All the analyses were performed using Epi-Info 7.0 (Centers for Disease Control and Prevention, CDC, Atlanta, GA, USA) and the SPSS Statistics version 20.0 (IBM Corp., Armonk, NY, USA).

## Results

Between January 2007 and December 2014, we identified 511 episodes of Kp BSI, 349 of which were caused by C-R Kp (68.3 %). These episodes occurred in 327 patients, of whom 19 had recurrent infections (5.8 %). The median age of patients was 68 years (interquartile range [IQR] 57–76). As many as 231/349 C-R Kp BSI occurred in males (66.2 %), thus defining a 2:1 male to female ratio. The median time to C-R Kp BSI diagnosis from hospital admission was 24 days (IQR 12–45), and the median length of hospital stay was 52 days (IQR 30–90).

During the study period, the overall incidence of C-R Kp BSI was 0.92/10,000 patient-days, increasing from 0.04/10,000 patient-days in 2007 to 1.77/10,000 patient-days in 2014 (Chi square for trend *p* < 0.001). Detailed year-by-year incidences of C-R Kp BSI are outlined in Table 1, which also reports annual incidences of C-R Kp BSI according to wards’ subgroups. As shown in the table, C-R Kp BSI were first observed in 2007, reflecting a limited cluster in ICUs. No C-R Kp BSI occurred in 2008, while they reappeared since 2009, with annual incidences increasing from 0.56/10,000 patient-days in 2009 to 1.75/10,000 patient-days in 2012. After a temporary decrease in 2013, the incidence of C-R Kp BSI rose again to 1.77/10,000 patient-days in 2014.

**Table 1:** Annual incidences of health-care associated carbapenem-resistant *Klebsiella pneumoniae* bloodstream infections

		Incidence per 10,000 patient-days (95 % CI)				
Year	No. of episodes	Ward where the diagnosis of C-R Kp BSI was made				
		Overall	ICU	Surgical wards	Medical wards	Rehabilitation wards
2007	2	0.04 (0.01 – 0.17)	1.15 (0.29 – 4.59)	-	-	-
2008	0	-	-	-	-	-
2009	27	0.56 (0.38 -0.82)	12.70 (8.44 – 19.1)	0.14 (0.04 – 0.57)	0.04 (0 – 0.28)	0.60 (0.09-4.27)
2010	39	0.83 (0.60-1.13)	17.18 (11.94 – 24.71)	0.08 (0.01 – 0.55)	0.32 (0.16 – 0.64)	0.44 (0.06 – 3.12)
2011	59	1.22 (0.94-1.57)	21.06 (15.40-28.81)	0.69 (0.36-1.32)	0.36 (0.19 – 0.69)	0.60 (0.02 – 2.39)
2012	83	1.75 (1.41-2.17)	22.01 (16.09 – 30.11)	1.55 (0.99-2.43)	0.96 (0.64 -1.44)	0.44 (0.11 – 1.76)
2013	60	1.30 (1.01 – 1.68)	18.17 (12.98 – 25.42)	0.61 (0.29 – 1.29)	0.72 (0.44 – 1.17)	0.48 (0.15 – 1.48)
2014	79	1.77 (1.42 – 2.20)	18.79 (13.62 – 25.93)	0.66 (0.31 – 1.37)	1.32 (0.92 – 1.89)	0.98 (0.41 – 2.36)

*C-R Kp BSI* carbapenem-resistant *Klebsiella pneumoniae* bloodstream infections, *ICU* intensive care unit

**Fig. 1:**
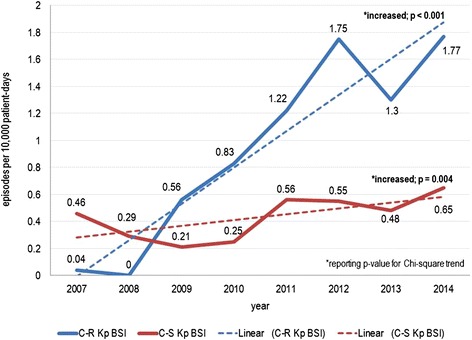
Annual incidences of health-care associated *Klebsiella pneumoniae* bloodstream infections (C-R and C-S)

Overall, the incidence of Kp BSI (C-R plus C-S) increased from 0.50/10,000 patient-days in 2007 to 2.42/10,000 patient-days in 2014 (Chi square for trend *p* < 0.001). The singular trends of C-R Kp BSI and C-S Kp BSI over the study period are depicted in Fig. 1. As shown in the figure, the overall increase in the incidence of Kp BSI was mostly due to C-R Kp BSI, although a less marked but significant increase of C-S Kp BSI was also observed (Chi square for trend *p* = 0.004).

Finally, crude 30-day mortality was higher in patients with C-R Kp BSI than in those with C-S Kp BSI (36.1 vs. 23.5 %, respectively). However, this comparison was not further considered in our analyses, since it could not be adjusted for some important clinical confounders (e.g., severity of illness, type of antibiotic therapy) that were not explored in this epidemiologic study.

## Discussion

From 2007 to 2014 we noticed an impressive increase of more than 300 % in the incidence of Kp BSI in our hospital, that was mostly driven by C-R strains. This finding is of particular concern, since C-R Kp BSI carry a heavy burden in terms of morbidity and mortality [3–6].

A worrisome and persistent increase of carbapenem-resistance among Kp isolates has been reported in Italy since 2005, with current national rates being as high as 34 % [11, 12]. Our experience confirms this picture, since we observed a rapid increase in the incidence of C-R Kp BSI starting from 2007, that peaked at 1.75/10,000 patient-days in 2012. A temporary decrease was then observed in 2013, possibly because of the intensification of our hospital-based infection control measures (i.e., rapid identification of colonized and/or infected patients trough a centralized and computerized laboratory-based alert system, patient dedicated use of gowns and gloves) that occurred at that time. Starting from the same period, an active surveillance of colonized patients and their contacts was also implemented, by means of rectal swab and according to European guidelines [9].

All these measures have been already proven to reduce the incidence of infections due to multidrug resistant gram negatives, including C-R Kp [15–19]. However, after the temporary decrease mentioned above, the incidence of C-R Kp BSI in our hospital rose again to 1.77/10,000 patient-days in 2014. Although a lack of adherence to contention measures might have occurred in some cases, another factor possibly underlie this increase that mostly occurred outside the ICU. Indeed, we recently observed that some C-R Kp intestinal carriers in low risk wards (i.e., wards other than ICU or cancer wards) might not be considered at risk (and thus not screened) even if all recommended infection-control protocols are adopted [20]. This finding suggests that a considerable number of unknown carriers might be circulating in our hospital that are not subjected to contact precautions because of the unawareness of their status. This possibility is rather worrisome, since it could lead to an uncontrolled C-R Kp diffusion, thus fueling the reservoir of patients who possibly develop C-R Kp BSI [20, 21]. In such an uncontrolled endemic setting, further infection-control efforts (e.g., a dynamic redefinition of wards to adopt universal screening through regular point-prevalence surveys) might be necessary to successfully counteract the alarming spread of C-R Kp.

The present study has some important limitations. First, we could not depict the true pattern of C-R Kp diffusion, since only blood C-R Kp isolates were considered. However, C-R Kp BSI can provide a reliable picture of this diffusion, since blood cultures are collected from nearly all patients with C-R Kp BSI because of the symptomatic nature of the infection. Conversely, any possible estimation based on colonized patients would have been affected by an important selection bias due to the differences in the number of screened patients across the study years. Second, the retrospective nature of the study prevented us from investigating the degree of compliance to infection-control measures during the study period, as well as the molecular characteristics of C-R Kp isolates. In this regard, a major limitation is the lack of information about both the clonal type of Kp isolates and their mechanisms of resistance to carbapenems. Finally, this was a single center study, and incidences of C-R Kp BSI may differ significantly across centers. Therefore, any extrapolation of our findings to other settings should be made with due caution.

## Conclusions

In our hospital a dramatic increase in the incidence of C-R Kp BSI occurred from 2007 to 2014 that appeared to be only temporarily arrested by the intensification of hospital-based infection-control measures. More concerted efforts are necessary to tackle this alarming C-R Kp diffusion. Because of the dramatic shortage in antibiotics active against C-R Kp, further improvements in our infection-control protocols are also of paramount importance to curtail the high number of deaths due to C-R Kp BSI.

## Abbreviations

BSI: Bloodstream infections
C-R: Carbapenem-resistant
C-S: Carbapenem-susceptible
CI: Confidence intervals
ECDC: European Center for Disease Control and prevention
EUCAST: European Committee on Antimicrobial Susceptibility Testing
Kp: *Klebsiella pneumoniae*
ICUs: Intensive care units
IQR: Interquartile range
MICs: Minimum inhibitory concentrations
